# How Do Communities Use a Participatory Public Health Approach to Build Resilience? The Los Angeles County Community Disaster Resilience Project

**DOI:** 10.3390/ijerph14101267

**Published:** 2017-10-21

**Authors:** Elizabeth Bromley, David P. Eisenman, Aizita Magana, Malcolm Williams, Biblia Kim, Michael McCreary, Anita Chandra, Kenneth B. Wells

**Affiliations:** 1 Department of Psychiatry and Biobehavioral Sciences, David Geffen School of Medicine at University of California, Los Angeles, CA 90095, USA; mmccreary@mednet.ucla.edu (M.M.); kwells@mednet.ucla.edu (K.B.W.); 2 West Los Angeles VA Healthcare Center, Los Angeles, CA 90073, USA; 3 Division of General Internal Medicine, David Geffen School of Medicine at UCLA; Los Angeles, CA 90095, USA; DEisenman@mednet.ucla.edu; 4 UCLA Fielding School of Public Health, Los Angeles, CA 90095, USA; 5 RAND Corporation, Santa Monica, CA 90401, USA; mwilliam@rand.org; 6 RAND Corporation, Arlington, VA 22202, USA; chandra@rand.org; 7 Los Angeles County Department of Public Health, 313 N Figueroa St, Los Angeles, CA 90012, USA; aimagana@ph.lacounty.gov; 8 School of Public Health, Loma Linda University, Loma Linda, CA 92350, USA; bskim@llu.edu

**Keywords:** community participatory methods, community resilience, disaster preparedness, community coalitions, public health nursing, disaster risk reduction

## Abstract

Community resilience is a key concept in the National Health Security Strategy that emphasizes development of multi-sector partnerships and equity through community engagement. Here, we describe the advancement of CR principles through community participatory methods in the Los Angeles County Community Disaster Resilience (LACCDR) initiative. LACCDR, an initiative led by the Los Angeles County Department of Public Health with academic partners, randomized 16 community coalitions to implement either an Enhanced Standard Preparedness or Community Resilience approach over 24 months. Facilitated by a public health nurse or community educator, coalitions comprised government agencies, community-focused organizations and community members. We used thematic analysis of data from focus groups (*n* = 5) and interviews (*n* = 6 coalition members; *n* = 16 facilitators) to compare coalitions’ strategies for operationalizing community resilience levers of change (engagement, partnership, self-sufficiency, education). We find that strategies that included bidirectional learning helped coalitions understand and adopt resilience principles. Strategies that operationalized community resilience levers in mutually reinforcing ways (e.g., disseminating information while strengthening partnerships) also secured commitment to resilience principles. We review additional challenges and successes in achieving cross-sector collaboration and engaging at-risk groups in the resilience versus preparedness coalitions. The LACCDR example can inform strategies for uptake and implementation of community resilience and uptake of the resilience concept and methods.

## 1. Introduction

The community resilience approach to disasters recognizes that the ways in which a community addresses emergencies must draw on enduring community resources and capacities [1]; and, moreover, that exposure to risk can sometimes result in community growth and development [2]. Community activities within the resilience framework consider long-term recovery from disasters, but also community strengths to manage other kinds of persistent and emergent threats, such as severe weather and hazardous exposures, as well as how impacts of these events can be exacerbated by community vulnerabilities, such as the number of at-risk individuals or low social connectedness [3]. Community resilience includes as a major goal improved capacity and information exchange in diverse communities.

Community participatory methods have provided a key strategy to bridge technical knowledge of preparedness to community contexts and stakeholders [4,5,6]. All approaches to building community resilience emphasize the need for linking community members, community-based organizations (e.g., churches, schools, nursing homes), businesses, and government agencies across sectors. For instance, the Sendai Framework for disaster risk reduction calls for “all-of-society engagement and partnership” with a focus on “inclusive, accessible, and non-discriminatory participation” and efforts toward “the improvement of organized voluntary work of citizens” [7] (p. 13). In the U.S. National Health Security Strategy (NHSS), community resilience entails enhancing preparedness through improving social connectedness and coordination between health and human services agencies [8]. The NHSS Implementation Plan also cites community participatory methods as an “inclusive, proactive approach” (p. 8) to building community resilience [9]. The United Nations International Strategy for Disaster Reduction (UNISDR) [10], the United States Federal Emergency Management Administration’s (2011) Whole Community Approach to Emergency Management, the United States Center for Disease Control and Prevention’s (2011) Community Resilience model, Environmental Public Health Performance standards [4,11], and the Rockefeller 100 Resilient Cities Initiative all emphasize the importance of community engagement and multi-sector partnerships [12] to integrate ethnically-diverse communities, and the organizations that serve them in emergency planning [13] to advance community resilience.

In this paper, we describe the advancement of community resilience principles through community participatory methods using a large mixed-method dataset. While many studies describe theories and definitions of community resilience [2,14,15], fewer describe the processes by which community stakeholders interpret and implement community resilience [16]. Most research reports do not include detailed empirical data to document real-world activities devised and directed by community stakeholders to advance community resilience. For instance, the Community and Regional Resilience Institute’s case studies provide considerable on-the-ground detail, but focus on institutional and programmatic efforts to anticipate, reduce, respond and recover from a disaster [17]. We use data from a community coalition-based disaster preparedness study supported by the Los Angeles County Department of Public Health. Through a randomized demonstration, the Los Angeles County Community Disaster Resilience (LACCDR) study compared enhanced standard preparedness and community resilience approaches in 16 community coalitions in diverse neighborhoods over three years. We use interview, focus group, and activity log data to describe community coalition members’ understanding of the community resilience concept and operationalization of its components; and to detail community-based activities chosen by coalition members to build resilience. We also review differences and similarities in activities between resilience and preparedness coalitions to suggest which aspects of the CR approach are readily adopted by community coalitions and which require enhanced support.

## 2. Materials and Methods

*LACCDR Intervention*: The LACCDR project was developed over two years by the Los Angeles County Department of Public Health working closely with community, academic, government and business partners [18,19]. A full description of study design, methods, coalitions, and the conceptual framework for LACCDR are published elsewhere [18,19,20,21]. Sixteen modest-sized (i.e., <50,000 persons) communities within Los Angeles County were identified and randomized to a novel community resilience program or the comparison program (Enhanced Standard Preparedness, ESP). Based on the work of Chandra and others, the community resilience concept was implemented through four pre-defined levers: engagement with community with particular attention to the needs of traditionally vulnerable populations, organizational partnerships specifically among government and nongovernmental organizations (NGOs), community self-sufficiency, and education and training through the disaster cycle [18].

The intervention models differed in their emphasis on community resilience, but shared a common focus on the development of community coalitions. Each coalition had a public health nurse or health educator assigned to facilitate (hereafter called a nurse facilitator). CR coalitions received training via a toolkit that included information on what community resilience is, how to focus on community assets, mapping hazards in the neighborhood, identifying vulnerable groups, recognizing and responding to psychological trauma, and how to build sustainability into community work (the toolkit and method is available at www.resilienceincommunities.com). ESP coalitions received training on traditional preparedness, special populations, and strategies for linking with community groups. While they did not receive specialized training in CR, ESP coalitions were exposed to some general aspects of the CR concept at annual overall project convenings and potentially through nurse facilitator expertise. Coalitions were instructed to meet monthly for two years, starting in February 2013. Each coalition received $15,000 to support their activities.

### 2.1. Data Sources

Activity Logs: Beginning in March 2014 through July 2015, each coalition’s nurse facilitator completed monthly activity logs to record meetings, trainings, and other coalition activities. We analyzed activity logs for June 2014 through May 2015, the 12-month period with minimal missing data. From the 16 coalitions over this 12-month period, we received 174 of the expected 192 activity logs (90.6%). Four study team members began by analyzing half of all activity log entries to identify patterns in activity type and develop a taxonomy of activities (i.e., extent of community engagement, target audience, sponsorship). We excluded from the taxonomy activities that coalitions conducted only with themselves, such as planning meetings. Two study team members applied and refined the taxonomy by coding the remainder of events, settling on four types of coalition activities (described below) that captured key features of all activities with communities.

Interviews and Focus Groups: The research team conducted phone interviews and in-person focus groups with coalitions between January and October 2015. All interviews and focus groups were conducted in English. Investigators invited eight coalitions to complete a focus group (three CR, five ESP) and five coalitions (four CR, one ESP) to complete interviews; alternative data sources were available to describe activities of the three remaining coalitions (one CR, two ESP). Two of the 13 invited coalitions did not respond to requests for either focus groups or interviews. Coalitions with sustained activities were more likely to complete interviews or focus groups. Six CR coalitions completed an interview (*n* = 4) or focus group (*n* = 2); and 5 ESP coalitions completed an interview (*n* = 2) or focus group (*n* = 3). 

Fifteen of sixteen nurse facilitators completed an interview between October and December 2015; the 16th completed an interview in March 2016. Interviews asked coalition members and nurse facilitators about coalition understanding of community resilience, coalition challenges and successes, coalition decision-making and goal setting, the role of organizational partners in coalition activities, and the role of the facilitator with coalitions. Interviews and focus groups lasted between 40 and 90 min and were audio recorded and professionally transcribed. 

Finally, between February and August 2015, the study team conducted approximately 6 h of interviewing with 3 investigators (Aizita Magana, Biblia Kim, Rachel Long) who described each coalition’s development and key activities. In this manner, at least two sources of data were available for all 16 coalitions.

### 2.2. Data Analysis

Data were coded in ATLAS.ti (ATLAS.ti Scientific Software Development GmbH, Berlin, Germany). Two coders used a grounded deductive content coding approach to mark passages that corresponded to levers of the CR concept (i.e., engagement, partnership, education, self-sufficiency). We generated summaries of activities related to each lever. Then, we created summaries of data in which levers co-occurred (e.g., education and engagement), identifying themes that described which levers co-occurred, how frequently, in what contexts. 

All data sources (i.e., study team interviews, nurse facilitator interviews, activity logs, coalition focus groups, and interviews) were triangulated to validate conclusions and identify disconfirming examples. In a member checking step [22], key study team members (David Eisenman, Anita Chandra, Kenneth B. Wells) reviewed preliminary findings and confirmed or augmented summaries of coalition activities

## 3. Results

### 3.1. Coalition Activities

Activity log analyses identified four types of coalition activities: fairs, events, outreach, and training. Fair activities were community events organized by an outside entity that coalition members used to increase awareness about disaster preparedness and/or resilience. Fairs were attended by large numbers (e.g., >100) of community members. Coalitions usually set up booths and had brief, low-touch contact with attendees. Events were activities in which coalition members attended a community disaster-focused event, sometimes co-organized by the coalition with a partner (e.g., the fire department), to bring awareness about preparedness and/or resilience. Compared to fairs, events involved more active, high-touch contact with a smaller number of community members. Outreach activities were coalition-organized or co-organized; they were aimed at reaching specific community groups (e.g., seniors) in order to increase preparedness and/or resilience; some outreach activities took place over many days, such as when coalition members distributed information to renters at multiple apartment complexes. Training activities were organized by coalition members to teach skills to community members; they provided cardiopulmonary resuscitation (CPR), Community Emergency Response Team (CERT), or other training content through traditional or train-the-trainer approaches.

Table 1 shows the frequency of coalition activities by study arm. The number of community-engaged activities varied considerably across coalitions, with total events ranging from two to 19. Both CR and ESP coalitions used outreach and events, but CR coalitions held more training activities (58 CR; 18 ESP) while ESP coalitions participated in more fairs (11 CR; 39 ESP). Both CR and ESP coalitions pursued activities focused on vulnerable groups (28 CR; 32 ESP). Activities focused on vulnerable groups were defined as those directed toward a subset of the community possessing a culture, language or other distinguishing characteristic that places them at higher risk in a disaster (e.g., school-aged children and their parents; ethnic minorities; homeless or food insecure populations). On average, between two and six coalition members (2.5 to 5.5) participated in each activity, with a slightly higher average number of CR coalition members (2.8–6.9) participating in activities of all type compared to the average number of ESP coalition members (2.4–4.7); these averages are based on small samples with high variation.

### 3.2. CR Understanding and Uptake

Qualitative data indicate that coalitions interpreted and implemented community resilience in diverse ways through these activities. We identified four distinct strategies used by coalitions to operationalize the four levers of community resilience (engagement with community, organizational partnerships, community self-sufficiency, and education and training) through community participation: (1) anchoring resilience in preparedness, (2) embracing diversity, particularly as a part of engagement, (3) engaging while educating about the disaster cycle, and (4) finding reciprocity in partnerships, among organizations. As described below, qualitative data sources suggest CR and ESP coalitions utilized these strategies to varying degrees and adopted priorities, activities, and language that frequently differed.

#### 3.2.1. Anchoring in Preparedness

While the community resilience concept describes preparedness as critical not just for disasters but also for persistent vulnerabilities (e.g., severe weather conditions, community violence), coalitions most frequently interpreted and implemented resilience through a focus on traditional disaster preparedness at the household level. As one CR coalition member said, “My definition of resilience is being prepared for when the disaster happens. That’s my personal definition.” Coalition members described issues like earthquakes, mudslides, or forest fires as concerns within their neighborhoods and natural starting points for engagement. As an ESP coalition member said, “People can relate to that.” Both ESP and CR coalitions, for instance, worked on issues of obtaining and ensuring availability of supplies in emergencies. Additionally, both ESP and CR coalitions emphasized the need for community members to be prepared for the absence of first responders after a disaster. One ESP coalition described their goal as “making sure that the people are aware that you are not going to be able to have the first responders come anytime soon.” One CR coalition developed a community medical response team that would triage community members requiring medical or psychological first aid in the event of a disaster when “we’re cut off; where we’re on our own.” 

Yet both CR and ESP coalitions described using disasters to focus on goals relevant to community resilience, including improving community collaboration and social cohesion. For instance, one ESP coalition sought to teach “individual preparedness but [also] how you tie in and motivate your organizations to be prepared.” One of this coalition’s members recognized the importance of the church: “I truly feel that in the event of a disaster…people will come to our church seeking assistance because they feel that the church is a safe place to go and they probably would feel that there would be some type of service…there available to them.” The importance of community leadership came into view as well: “I knew that as a church leader, I would be responsible or looked to provide leadership.” An ESP coalition member recognized the importance of relating more regularly within communities: “I hope that people want to really talk to their neighbors about it, learn their neighbors, because a lot of us like even me…I don’t go to my homeowner meetings…I gotta do more than that, I gotta really get involved.” 

Some coalitions pursued novel community resilience-informed goals with minimal relationship to traditional disaster preparedness. One CR coalition identified a growing interest in community safety when community leaders who had been passionate about safety brought their concerns to the coalition. A CR coalition transitioned from an initial focus on household preparedness topics (e.g., household emergency supplies) to a broader focus on communication to entire neighborhoods, training new amateur radio operators, and purchasing an electronic billboard to announce community events. A CR coalition and an ESP coalition built on an existing network of amateur radio operators to improve community connections. One ESP coalition prepared workshops on sustainable energy and a second CR coalition prepared a symposium on drought and water conservation.

#### 3.2.2. Embracing Diversity

The engagement lever of community resilience emphasizes the goal of generating participation from a diverse set of community members. Coalitions discussed diversity in various ways, such as attending to vulnerable community members, engaging many sectors, and representing ethnic communities. As one CR coalition member said, their “greatest strength is the diversity of the members in the coalition.” For instance, one ESP coalition co-sponsored a fire safety event and, “there are more of the Chinese native speaking population…who attended as well as the Latino non-English speaking communities; and (their attendance) was strictly the result of our cooperation.” One of the most common ways that coalitions increased diversity was by embracing diverse languages in activities and materials. Since language skills needed to be found within the community, not all coalitions supported language diversity to the same extent. ESP coalitions mentioned the importance of multi-lingual training manuals, but not all coalitions forged ties with those communities. As the facilitator said of one ESP coalition, “unfortunately we didn’t have that representation (of other language speakers) on our coalition. I really wish that we had worked harder to get that but then we didn’t really have those connections to bring people in.” In contrast, one CR coalition supported language diversity using translation equipment and language proficiency among coalition members to translate training and coalition meetings into Spanish and Korean. As the nurse facilitator said, as the coalition developed, “they started inviting more youth, more Korean speaking, more ethnicities; they actually welcomed a lot more people later on.” 

Not all coalitions invested equally in increasing diversity. One CR coalition pursued an intentional and persistent strategy to increase diversity. Coalition members described extensive efforts to improve coalition diversity. They divided their community into geographical and occupational sectors and reached out to establish contacts in each sector using a survey tool and were successful in reaching diverse groups: “We found some elderly people with health needs, we found some people with young kids, I found a day care center in our neighborhood that I didn’t know existed.” Yet coalition members still recognized that they could be criticized for being too “white Anglo-Saxon protestant,” while the community was highly diverse, particularly because it included a large non-English speaking Korean community. According to the nurse facilitator and coalition members in the focus group, the coalition members were uncertain how to engage this community. They said both that diverse groups may have been reluctant to join and, as one said, “we didn’t fight to get them there either.” Yet the nurse facilitator was able to improve coalition members’ readiness. When the nurse facilitator noticed at a fair that members of the Korean community were approaching their booth but not speaking to coalition members, “I started engaging with them and I said ‘hey come here,’ and you know, I’m a veteran at it, so I started engaging” with the use of translated materials. She continued: “I said ‘bring your friends’; all of a sudden the booth was full with about twenty other people” picking up materials. Her coalition learned from her approach: “all the coalition members were so impressed, they were like ‘wow’ and then they started doing it.”

Compared to CR coalitions, ESP coalitions pursued a more limited approach to increasing diversity. One ESP coalition member said, “I met somebody today involved with the Hispanic community, which is most helpful to us … So that was a bridge that has been leapt and we’re now able to make contact with them to broaden our expertise.” Another ESP coalition said, when commenting on the diversity of the coalition, “I would like to have more ethnic (representation). I would like to bring in Latino, African American, you know, make it more diverse.” This member described “reaching the ethnic groups” as the “worst” and “most difficult” aspect of their coalition work, “but you do what you can and if they participate great, but if they don’t, that’s all you can do.” One ESP coalition indicated that, to continue to develop their work, they would not need to invite any new members or link to any other groups. They discussed a strong intention to “continue along the same process, try to improve it in certain ways, streamline it,” rather than expand to include new members and new ideas. Of this coalition, the facilitator said, “I had always kind of thought or hoped that one, they would bring in more community partners in the coalition.” She continued, “nobody ever really went out and recruited other sectors (like schools or businesses), public sectors, and brought them into the coalition.” 

#### 3.2.3. Engaging While Educating

The community resilience lever of education affects change in community through information exchange about preparation, risks, and resources before, during, and after a disaster. As shown in Table 1, ESP coalitions most commonly used fairs to conduct education in their neighborhoods. For instance, an ESP coalition hosted a city-wide emergency preparedness event with representation from fire, police, emergency management, and vendors with an estimated 500 attendees. Many ESP coalition members talked about education as a one-way exchange. As one said, “I think the information is there, it’s just a matter of people finding a way to get it.” As one member said, “any place I go where there are brochures or anything, I’ll try and get as many of them as I can and I take them back” to others. Another ESP coalition created a new brochure with information specific to their city. A fourth ESP coalition focused on “shar(ing) (preparedness information) with people; so we went to senior parks, and in their day rooms we would share what we learned” related to preparedness. 

Three ESP facilitators expressed a desire that their coalitions had more actively engaged the community in educational activities. For instance, the facilitator of one ESP coalition said they did “outreach events just giving out information on emergency preparedness, so very traditional one-on-one education, quick highlights of things. And that’s kind of what they limited their role to the very end.” An ESP facilitator said, during emergency fairs, “I would just, mainly, man the table” because coalition members would not assume this task and “people would just walk up and take everything off the table.” The facilitator of another ESP coalition (ESP2) encouraged her coalition to expand their educational goals so that after teaching preparedness modules coalition members would “actually get out there and work with the community.” Nonetheless, several ESP coalitions used trainings effectively. One ESP coalition held CPR classes for kindergarten teachers, made presentations with the Red Cross to students, and attended fairs and festivals, including one in which the coalition gave information to 300 individuals who stopped by their booth. Another ESP coalition reached seniors by holding a two-hour Brown Book Training event at a Senior Center and conducted trainings for an inter-faith group at a local church, at schools, and at a mobile home park. 

In contrast, interview or focus group data from seven of eight CR coalitions consistently described education and engagement as synergistic goal that were best pursued simultaneously. Several CR coalitions described educational events as equal parts engagement and information exchange. According to members’ descriptions, every event held by one CR coalition (CR2) added coalition members, built capacity, and allowed new community members to shape subsequent activities. Trainings for high school students, at an elementary school, a middle school, and with the Conservation Corp used a train-the-trainer model that encouraged trainees to lead subsequent trainings. They conducted outreach at an elementary school fair, partnered with a police department for their open house, and held refresher courses for trainings. At these events, “we brought in some community folks that were part of the coalition that weren’t able to come to every meeting” and as a result of every training, “there’s a new group” interested in joining coalition meetings. Similarly, another CR coalition conducted trainings with senior groups, organizations for the disabled, and the civic and faith sectors with the goal of engaging them to join the coalition. Another CR coalition facilitator described her coalition’s trainings at an elementary school as prelude “to continue with that relationship, continue seeing them and sharing and try to empower them with information and resources.” Another CR coalition organized a community potluck and invited first responders and guest speakers to educate neighborhood members about preparedness. It was a success, according to the facilitator, because “kids were running around, people were eating, they had the mic on, they had music, it was a big party but at the same time everybody was paying attention” to the “good information” shared there. 

Only CR coalitions discussed the importance of learning from communities rather than distributing information to them. As one nurse facilitator described their coalition focus, “we have to build around the community needs and not the other way around.” One CR coalition said held an open community meeting at the start of their process and learned that communication in the event of a disaster was a key community concern. As a result, “we kind of got a sense of, well, we need accurate information out there so that they can kind of tell what’s going on,” and focused on this issue, such as by conducting amateur radio training. Another CR coalition described the need for a process of community feedback and buy-in before deciding how to spend study funds. In another CR coalition, the coalition worked to “tailor” their trainings toward community needs and preferences, conducting more CPR trainings because, as the facilitator said, CPR training was “something tangible that people feel empowered after completion.” As the facilitator said, “over time it kind of became a little more focused on what activities the community (felt) can really have meaningful impact.”

#### 3.2.4. Reciprocity in Partnerships

The lever of organizational partnerships entails building robust relationships within and between communities and governmental and non-governmental organizations through trust-building, reciprocity, and mutual exchange. Both CR and ESP coalitions understood the importance of these partnerships. Most coalitions described efforts to recruit new partners, ensure their attendance at coalition meetings and events, and find opportunities to align their interests with those of the coalition. As one CR coalition member said, “you really need to get the city officials, councilmen, law enforcement, the mayor’s office, fully invested from the get go. We used to have our city councilmen invested, and a bit of our mayor’s office, (police department) invested, but I think our job now as we move forward is to get them fully invested.” Doing so, as this ESP coalition recognized, was key to making the project a success: “It became the city’s activity, not just our coalition’s activity.” 

Coalition members described forging different types of relationships with partners. Partnership descriptions from ESP coalitions usually described transactional relationships, such as receiving resources from partners that bolstered their activities. An ESP coalition said that as a result of a city partnership, “our advertising [was] a lot better….We got it on the newspaper, we got it on the (city) website.” The city donated or paid for rental tables, chairs, and music, and city officials made presentations. Another ESP coalition nurse facilitator described similar support from the city, such as offering time at city events and loaning facilities to the coalition free of charge. Non-governmental partners also contributed resources, for example in another ESP coalition in which local stores set up disaster preparedness displays to encourage community members to stock up on flashlights, batteries, and other preparedness supplies. Another ESP coalition cited that their neighborhood council made a geologist available to them to educate the coalition about neighborhood hazards. 

In contrast the CR coalition members described that resources moved in both ways within partner relationships more often than ESP coalition members. For instance, one CR coalition trained Neighborhood Watch partners in CPR using a train-the-trainer model so that they could then train their neighborhood members. Another CR coalition supported partners’ activities, such as by helping to advertise for a partner-sponsored blood drive. The coalition also worked with partners, including the fire department, public works, and the water district, to identify a flood hazard in their neighborhood and to develop a solution. The coalition recognized that “it was the relationships between the community members” and the public works and water departments that “brought the problem to their attention” and led to the result that “they sat down, they figured it out.” Some ESP coalitions also identified the value of reciprocity. For this ESP coalition “a lot of what we did originally amongst our own [partners] was favor-for-a-favor. It’s like, none of us really have money to do this, but if you come and help me put on this…presentation, I’m gonna come and help you do this community fair. And we’ll share resources and people and information, and that’s how we’re gonna extend our reach.” 

Both ESP and CR coalitions commented that growth in coalition partnerships was not linear. Coalitions said they came to understand that partners were engaged even if they could not attend meetings. One ESP coalition said that partners in business, healthcare, school, and city sectors “have not been showing up… but when we yell for them, they show up. They still support us.” In fact, “just the other day,” a representative of the business sector “came to us and asked us to have a booth at their community outreach.” Another CR coalition said it was key that “you stick with those people, you nurture them and get them involved, you participate in other community events, you give back to them when they need something” because “those are the people that are going to make a difference when a disaster happens.” Another ESP coalition described as their aim the metaphor of a three-prong cord involving government, community organizations, and community members that “is not easily broken.” A coalition member saw partnerships as a way to take the first step in repairing the “disconnect between the community and the government agencies.” As he said, the coalition “has developed into being a trusted resource and link between the government who wanted to extend services and the community who needed it.”

## 4. Discussion

In this paper, we review data from 16 coalitions implementing community resilience in a randomized demonstration in Los Angeles County. The demonstration utilized a framework of community resilience comprising the theoretical levers of education, engagement, partnership, and self-sufficiency. Our data suggest that community coalition members understood and implemented these levers through strategies that often still anchored resilience in preparedness, embraced diversity, engaged community members while educating, and built reciprocity in partnerships. In other words, coalitions often conceptualized the lever of self-sufficiency in a traditional preparedness manner, such as in the context of a natural disaster when “we’re cut off; where we’re on our own.” They understood and implemented partnership as a task of developing give-and-take and identifying mutual benefit across sectors. To operationalize engagement, coalitions embraced diversity and used educational activities as a means of bringing new groups and community members to the table. Coalitions’ choices of strategies illustrate the ways in which communities may interpret and implement the resilience concept to build on local resources, preferences, and priorities. Since the strategies emerged from the deliberation of diverse real-world coalitions, they represent community-driven solutions for transforming traditional preparedness activities into broader, more ambitious efforts to bolster resilience. 

Community coalitions in the ESP and CR conditions implemented different strategies in qualitatively distinct ways, with CR coalitions out-performing ESP coalitions in the use of the levers of community resilience. For instance, ESP coalition relied more on fairs and low-touch events (11 vs. 39) while CR coalitions utilized high-touch trainings (58 vs. 18) and sponsored five times as many trainings to reach vulnerable groups (20 vs. 4) (Table 2). Compared to CR coalitions, ESP coalitions pursued a more limited approach to increasing diversity, though reaching diverse communities was difficult for both types of coalitions, suggesting this aspect of requires persistent support. Both ESP and CR coalitions embraced levers of education, engagement, partnership, and self-sufficiency; but these levers were not always employed synergistically in ESP coalitions, while seven of eight CR coalitions described education and engagement as goals to be pursued simultaneously. More actively than ESP coalitions, CR coalitions sought reciprocal relationships with partners, looking to achieve mutual gains; other data from LACCDR show that, compared to ESP coalitions, CR coalitions did more partnership building with more diverse sectors. Further, only CR coalitions mentioned the importance of learning from communities—rather than distributing information to communities—during educational activities. In these ways, CR coalitions were more focused on engagement as a process of learning *and* teaching, a feature that distinguishes the community resilience approach from the more top-down traditional preparedness approach. 

Coalitions’ use of these four strategies provides lessons for community resilience implementation. For instance, community resilience anchored in preparedness meant that, even when well-versed in the community resilience concept, coalitions perceived that community members would resonate with goals focused on anticipating, preparing for, and recovering from disasters. Some coalitions built on these disaster-related goals to attend to issues more unique to building resilience instead of just hazard preparedness, like community cohesion or response to pervading challenges, like drought. Anchoring resilience within preparedness allowed coalitions to work on high-profile and dramatic community problems while working toward more challenging goals like overall community strength and proactive management of community vulnerabilities. This indicates that resilience priorities need not replace but can build on traditional preparedness concerns. It may be that coalitions built on preparedness strengths and personnel because they were less familiar with the tenets of community participation and community resilience, such as engaging diverse community members, seeking input on goals, and working to improve social connectedness. This would suggest that having an intentional approach that teaches community resilience values over and above preparedness may be key to building capacity to extend beyond traditional preparedness tasks. 

All coalitions embraced the idea of diversity through inclusion of various sectors, diverse ethnic community members, and various languages, but achieving diversity was a continual challenge. Encouragement and modeling of engagement techniques helped coalitions make progress. All coalitions faced challenges finding language skills to facilitate translation of materials and meetings. These findings suggest that providing language resources could be a key support for diversity in coalitions. Similarly, data suggest that, with appropriate supports, coalitions are able to leverage educational events, like training sessions, to engage with new community groups. These supports included a nurse facilitator trained in community engagement, but may also include partnering with an agency that specializes in engagement of diverse populations. Without training resources, however, some coalitions defaulted to passive information exchange educational models that disseminated information but did not necessarily bring new community members or groups to the project. We suggest that community resilience activities should include persistent efforts to couple activities around education and engagement, such as by using train-the-trainer models and providing all trainees with low-demand options for staying involved in CR-building efforts. 

Coalition members suggested that partnerships could be key supports for coalition activities, but that true reciprocity in partnerships—in which resources and learning moved bi-directionally—maximized success. This indicates that resilience approaches may require a longer time course and heavy investment in relationship-building. It seemed to help coalitions to realize that community resilience methods need not be implemented in a linear fashion or with optimal participation from day one. As coalitions developed, they recognized the value of partners that might come “on and off the bus” to support resilience-building work [23]. The importance of passion for community wellbeing and of the nurturance of relationships were some of the fundamental lessons coalitions cited. A future publication drawing on additional data from LACCDR leaders will describe the role of factors like commitment to the community in supporting the sustainability of coalition activities beyond the timeline of the LACCDR project. Coalitions that expected for the work to evolve weathered the ups and downs of resilience-building best.

Overall, these findings provide evidence for the strong synergy between community participatory methods and the advancement of community resilience [24,25,26]. In fact, both CR and ESP coalitions found participatory methods and mindsets to be helpful. Despite little encouragement to do so, ESP coalitions embraced diversity and worked to build reciprocal partnerships, goals consistent with the Sendai Framework and other participatory approaches. One ESP coalition even articulated its mission as building a trusted bridge between government and community, as participatory projects often aim to do. Broadly speaking, participatory methods prioritize learning from local communities, seeking out a diversity of opinions, modifying goals as participants recognize their needs, focusing on community strengths, and sharing decision-making power. Coalition strategies for implementing the resilience levers, such as aiming for reciprocity, seeking diversity, and finding mutually-reinforcing goals, like educating while engaging, reflect the priorities of community participatory approaches. Many coalition members sought common points of understanding to develop shared agendas across diverse collaborations. They also shared resources to extend their reach, sought to continue relationships over time regardless of the terms, and they considered which supports (e.g., translation) would be needed to advance equity. These activities reflect the ways in which priorities and tactics adopted in participatory approaches may support resilience-building program implementation [26,27]. 

This study has limitations, including potential undercounting of some coalitions’ activities due to missing activity logs and distinct qualitative data collection approaches across coalitions (i.e., focus group vs. interviewing), which may overstate differences by generating more data from the most engaged and self-sustaining coalitions. Data collection was conducted in English and thus may have not included the range of perspectives. Since interviews were conducted by project sponsors, coalition members and facilitators may have shared less information about challenges than about successes; a future publication will describe coalitions’ challenges in more detail. The real-world setting of the LACCDR demonstration project likely blunted the comparisons that can be attributed to the study arm (ESP vs. CR). At the time of its design, few guidelines were available to shape implementation of a project like LACCDR, and LACCDR investigators were both creating a program while implementing and evaluating it. This innovative aspect of LACCDR resulted in some delays and inconsistencies in implementation—thereby increasing differences between coalitions—as well as gaps in evaluation metrics.

Moreover, qualitative descriptions of coalition approaches did not always reflect counts represented in activity logs. For instance, ESP coalition members may have described less active efforts to build diversity, yet activity counts indicate they reached more community members, particularly because of their use of fairs. Moreover, some coalitions that reported a small number of activities (e.g., CR2) nonetheless described their engagement with community as robust and evolving. These findings may reflect data limitations, but they may also suggest that coalition activities to build resilience ways have looked thin compared to other coalitions but may have been appropriate for the local context. Finally, nurse facilitators for CR coalitions were very experienced in preparedness but not in community participation, while nurse facilitators for ESP were familiar with community participation but new to the preparedness focus. This may have shaped ESP nurse facilitator perceptions that ESP coalitions could have pursued more active engagement, as well as CR nurse facilitator perceptions of the importance of standard preparedness. A future study design would ideally allow for building on the strengths of both approaches. 

## 5. Conclusions

LACCDR coalitions implemented community resilience by anchoring it in preparedness, embracing diversity, engaging while educating, and building reciprocity in partnerships. CR coalitions more often utilized high-touch activities that allowed for bidirectional learning from communities and partner organizations and, as a result, adopted strategies that foregrounded and capitalized on the synergistic potential of theoretical levers of community resilience (education, engagement, partnership, and self-sufficiency). Ongoing support for extending diversity and for finding opportunities for bi-directional learning and resource-sharing should be integrated into CR initiatives. Detailed data such as these from real-world initiatives can inform effective and sustainable use of community participatory methods to support implementation of community resilience.

## Figures and Tables

**Table 1:** Coalition activities by intervention status.

Coalition	All Activities	Activity Types				Activities for Vulnerable Population				
		Fair	Event	Outreach	Training	Total	Fair	Event	Outreach	Training
CR3	3	1	1	0	1	0	0	0	0	0
CR4	19	3	0	0	16	1	1	0	0	0
CR6	12	2	0	0	10	6	0	0	0	6
CR5	9	2	5	1	1	5	1	2	1	1
CR2	2	0	1	1	0	2	0	1	1	0
CR8	4	0	0	1	3	2	0	0	1	1
CR7	15	2	0	1	12	1	0	0	0	1
CR1	19	1	2	1	15	11	0	0	0	11
ESP4	8	4	1	1	2	5	2	0	1	2
ESP1	9	5	0	0	4	5	3	0	0	2
ESP2	9	5	0	2	2	3	1	0	2	0
ESP3	4	1	3	0	0	0	0	0	0	0
ESP7	6	1	1	2	2	3	0	1	2	0
ESP8	8	3	1	2	2	1	1	0	0	0
ESP6	19	11	1	1	6	5	4	1	0	0
ESP5	15	9	2	4	0	10	5	1	4	0
Total CR	83	11	9	5	58	28	2	3	3	20
Total ESP	78	39	9	12	18	32	16	3	9	4
Total	161	50	18	17	76	60	18	6	12	24

**Table 2:** Community resilience-building strategies.

Strategy	Description
Anchoring in preparedness	“(Our goal is) making sure that the community is aware of what their options are to prepare for an eventual emergency…and to know what things they need, where they can get them, and then who they can talk to after we have a disaster.”
Embracing diversity	“(We were) able to incorporate other minority groups that would typically not attend.”
Engaging while educating	“We’re going to get them involved in the medical training and in that way get them more invested in the coalition in general.”
Reciprocity in partnerships	“I think we’re very fortunate that we got to work with the city…because they were invested and they wanted to learn—they wanted to be part of the plan.”
